# Accuracy of CBCT, Digital Radiography and Cross-Sectioning for the Evaluation of Mandibular Incisor Root Canals

**DOI:** 10.7508/iej.2016.02.006

**Published:** 2016-03-20

**Authors:** Hadi Assadian, Arash Dabbaghi, Morteza Gooran, Behrouz Eftekhar, Sanaz Sharifi, Nassim Shams, Ali Dehghani Najvani, Hamed Tabesh

**Affiliations:** a *Department of Endodontics, Dental School, Shahed University, Tehran, Iran; *; b *Department of Oral and Maxillofacial Radiology, Dental School, Ahvaz Jundishapur University of Medical Sciences, Ahvaz, Iran; *; c *Department of Orthodontics, Dental School, Ahvaz Jundishapur University of Medical Sciences, Ahvaz, Iran; *; d *Department of Endodontics, Dental School, Ahvaz Jundishapur University of Medical Sciences, Ahvaz, Iran; *; e *Department of Oral and Maxillofacial Pathology, Dental School, Ahvaz Jundishapur University of Medical Sciences, Ahvaz, Iran; *; f *Department of Biostatistics and Epidemiology, Faculty of Health, Ahvaz JundiShapur University of Medical Sciences, Ahvaz, Iran*

**Keywords:** Anatomy, Cone-Beam Computed Tomography, Digital Radiography, Incisor Teeth

## Abstract

**Introduction:**

The aim of this study was to compare the accuracy of cone-beam computed tomography (CBCT), digital radiography and tooth sectioning in evaluating root canal morphology of mandibular incisors in an *in vitro* setting.

**Methods and Materials:**

A total of 76 samples were imaged using CBCT, and digital radiography in straight and angled views. The samples were then sectioned at different distances from the apex for further visualization under stereomicroscope. The agreement between the observers was statistically analyzed by kappa correlation coefficient and the chi-square test.

**Results:**

The results showed that the majority of the samples had a single canal (Vertucci’s *Type I*). CBCT analysis reported more frequent multi-canal roots in comparison with the other techniques. In pairwise comparisons, the highest agreement was found between digital radiographic imaging and microscopic cross-sectioning both in terms of canal configuration and the number of root canals.

**Conclusion:**

None of the used imaging techniques *per se* could adequately show the exact internal anatomical configuration in accordance with the gold standard.

## Introduction

Successful endodontic treatment relies upon a thorough knowledge about the internal anatomy of the teeth. Such information is also of anthropological importance due to the variations observed in different races [1]. Regarding the genetic influences on the dental anatomy, different anatomical studies are necessary in different racial groups.

Several methods have been proposed to study the internal anatomy of the root canal system including root canal staining and clearing [2, 3], conventional, digital and contrast-medium enhanced radiography [4-6], and various types of computed tomography [7-9]. None of the aforementioned techniques can be addressed as the gold standard for evaluation of the internal dental anatomy due to their inherent drawbacks [1].

Studies on internal anatomical details are frequently found about mandibular [10] and more specifically maxillary molars and premolars [1, 2, 11-13], whereas anatomical variability within the root canal system of the mandibular incisors cannot be overlooked. Mandibular incisors usually have a single root containing a long narrow canal [14]. Often, a dentinal bridge within the pulp space creates two canals within the single root [15]. These two canals merge together to end in a single apical foramen, but there is a possibility for them to be separated from each other all throughout their pathway [16, 17]. Sometimes inability to thoroughly clean, shape and obturate the lingual canal causes failure of endodontic treatment. 

Cone-beam computed tomography (CBCT), uses a fan-shaped radiation source that creates three-dimensional images by a 180-360 degree rotation and offers a reduced patient exposure in comparison with conventional computed tomography. In addition, another superiority of the resultant image is the lack of structural superimpositions [18]. Capability of this technique in detection of vertical root fracture [19], measuring the root canal length [20], determination of the root canal curvature [21], evaluation of root canal changes following instrumentation [22] and also evaluating the second mesiobuccal canal of the maxillary first molars [11, 23] has also been studied.

Although morphological studies using CBCT are numerous, to the best of our knowledge, there is no available study comparing the diagnostic capability of this technique with digital radiography and microscopically evaluated cross-sections, especially in mandibular incisors. Therefore, the aim of this *in vitro* study was to compare CBCT, digital radiography and microscopically-evaluated cross-sections in evaluation of root canal morphology of human mandibular incisors.

## Materials and Methods

In this study, 76 freshly extracted mandibular incisors obtained from southern Iranian population without any cracks, fractures or external root resorption were selected. Teeth were randomly divided into 19 groups (*n*=4) for imaging convenience. The roots were immersed into melted wax to simulate periodontal ligament space. Then, the samples were mounted in acrylic blocks. In order to simulate bone trabeculation, dried and powdered sheep skull was equally mixed with acrylic resin powder before preparation of the acrylic blocks.

***CBCT imaging***

The peripheral part of each sample was covered with four layers of rose wax to simulate soft tissue. CBCT scans were carried out using Planmeca Promax 3D (Planmeca, Helsinki, Finland). The images were taken at 84 kVp, 6 mA and 12-sec exposure. The field of view (FOV) was set at 5×5 cm with the pixel size of 0.16 mm and bit depth of 15. The images were analyzed by Planmeca Romexis Viewer (Romexis software version 2.8.1) (Planmeca OY, Helsinki, Finland) using a 17-inch monitor (L1752SE Series, LG Corporations, South Korea) with a resolution of 1280×1024 pixels in a dark room. Brightness and contrast for each image was adjusted for better visualization. The images were precisely evaluated by three independent oral and maxillofacial radiologists. The images were visualized under similar conditions as described above to ensure evaluators’ calibration.

***Digital radiographic imaging***

After mounting the teeth, digital radiographies were taken using a photostimulable phosphor plate, (Digora, Soredex, Orion Corporation Ltd., Helsinki, Finland) at 70 kVp and 8 mA. Each sample was subjected to a straight (with 0^°^ horizontal and 0^°^ vertical tube angulations) and an angled radiography (with +20^°^ mesially angled horizontal and 0^°^ vertical tube angulations). Images were visualized by Scanora (Soredex, Helsinki, Finland) using the aforementioned monitor under similar circumstances. The straight and mesially-shifted radiographies were visualized by three independent endodontists, at least three times for each observer to reduce intra-observer error. Image enhancement option was also used to improve diagnostic ability. The radiographies were also visualized under standardized conditions to ensure evaluators’ calibration. 

**Table 1:** Frequency of different morphological types according to Vertucci’s classification observed by different methods

	Digital Radiography N (%)	Microscopic Evaluation N (%)	CBCT N (%)
Type I	40 (52.6)	53 (69.7)	33 (43.4)
Type II	1 (1.3)	4 (5.3)	4 (5.3)
Type III	24 (31.6)	19 (25)	38 (50)
Type IV	0 (0)	0 (0)	0 (0)
Type V	11 (14.5)	0 (0)	1 (1.3)

**Table 2:** Agreement crosstab between digital radiographic imaging and CBCT groups in terms of canal counts

	CBCT N (%)				
		Type I	Type II	Type III	Type IV
Digital Radiography N (%)	Type I	27 (35.5)	13 (17.1)	0 (0)	0 (0)
	Type II	6 (7.9)	29 (38.2)	0 (0)	0 (0)
	Type III	0 (0)	0 (0)	0 (0)	0 (0)
	Type IV	0	1 (1.3)	0 (0)	0 (0)

**Table 3:** Agreement crosstab regarding internal anatomy of the specimens according to Vertucci’s classification between different techniques

	CBCT N (%)					
		Type I	Type II	Type III	Type IV	Type V
Microscopic Evaluation N (%)	Type I	30 (39.5)	0 (0)	23 (30.3)	0 (0)	0 (0)
	Type II	2 (2.6)	1 (1.3)	1 (1.3)	0 (0)	0 (0)
	Type III	1 (1.3)	3 (3.9)	14 (18.4)	0 (0)	1 (1.3)
	Type IV	0 (0)	0 (0)	0 (0)	0 (0)	0 (0)
	Type V	0 (0)	0 (0)	0 (0)	0 (0)	0 (0)

***Microscopic evaluation of cross-sections***

After removing the samples from the acrylic blocks, they were mounted in a liquid adhesive. The teeth were decoronated to reach an average root length of 12 mm. The samples were then sectioned at 6 equal distances from the apex to create 2 mm-thick slices from each sample, using a saw microtome (Leitz 1600 Saw operative dentistry Microtome, Ernst Leitz, Wetzler, Germany). The sections were visualized by an oral and maxillofacial pathologist under a stereomicroscope (Zeiss DSM 940A, Carl Zeiss Inc., Oberkochen, Germany) under 40× magnification. All observers were blinded to each other’s visualizations. 

The data gained through either of the aforementioned techniques, were recorded according to Vertucci’s classification [15, 24]. The agreement between the observers was statistically analyzed by kappa coefficient.

## Results

A total of 76 samples were evaluated in this study. All three methods of evaluation showed that the majority of the samples had a single canal with *Type I* configuration according to Vertucci’s classification, except for CBCT imaging which indicated Vertucci’s *Type III* to be the most frequent anatomical configuration (Table 1). CBCT viewers reported more frequent multi-canal observations in comparison with those using other techniques. The results of the root canal configurations are shown in Table 1 for each method of evaluation. In addition, the agreement between the methods of evaluation in terms of canal configuration according to the Vertucci’s configuration system as well as the canal counts are shown in Tables 2 to 5. In general, according to the kappa statistical test, the agreement between CBCT and digital radiographic imaging observers in canal configuration was 0.48. On the other hand, in terms of canal configuration based on Vertucci’s classifications, the agreement between CBCT and digital radiographic imaging with cross-sections was 0.420 (*P*=0.001) and 0.618 (*P*=0.026), respectively. 

Totally, in terms of canal counts, the agreement between CBCT and cross-sections was 0.500 (*P*=0.28). Also, the agreement between digital radiographic imaging and cross-section was calculated to be 0.657 according to the kappa statistics (*P*=0.004). The agreement of the CBCT and cross-section methods in detection of root canals classified as Vertucci’s *Type I* and non-*Type I* configurations was 0.18 according to the kappa statistics. In addition, the agreement between digital radiographic imaging and cross-sections methods in diagnosing root canals classified as Vertucci’s *Type I* and non-*Type I* configurations was calculated to be 0.309 using kappa statistics. Because of the low value of kappa coefficient, sensitivity and specificity of the digital radiographic imaging and CBCT methods were calculated in comparison with cross-sections method. Accordingly, the sensitivity and specificity of the CBCT in comparison with cross-section method was 0.340 and 0.913, respectively; and the area under curve was calculated to be 0.626. The sensitivity and specificity of digital radiographic imaging compared with cross-section method was found to be 0.623 and 0.739, respectively; and the area under curve for this calculation was found to be 0.681.

## Discussion

The aim of this study was to compare CBCT, digital photostimulable phosphor plate radiography and microscopic cross-sectioning in evaluation of root canal morphology of human mandibular incisors in an *in vitro* setting. The samples included freshly extracted mandibular incisors obtained from a Southern Iranian population. 

**Table 4:** Agreement crosstab regarding internal anatomy of the specimens according to Vertucci’s classification between digital radiographic imaging and cone-beam computed tomography

	CBCT N (%)					
		Type I	Type II	Type III	Type IV	Type V
Digital Radiography N (%)	Type I	27 (35.5)	1 (1.3)	12 (15.8)	0 (0)	0 (0)
	Type II	0 (0)	0 (0)	1 (1.3)	0 (0)	0 (0)
	Type III	3 (3.9)	1 (1.3)	19 (25)	0 (0)	1 (1.3)
	Type IV	0 (0)	0 (0)	0 (0)	0 (0)	0 (0)
	Type V	3 (3.9)	2 (2.6)	6 (7.9)	0 (0)	0 (0)

**Table 5:** Agreement crosstab regarding internal anatomy of the specimens according to Vertucci’s classification between digital radiographic imaging and microscopically evaluated cross-sections

	Digital Radiographic Imaging N (%)					
		Type I	Type II	Type III	Type IV	Type V
Cross-sections N (%)	Type I	34 (44.7)	0 (0)	12 (15.8)	0 (0)	7 (9.2)
	Type II	2 (2.6)	0 (0)	1 (1.3)	0 (0)	1 (1.3)
	Type III	4 (5.3)	1 (1.3)	11 (14.5)	0 (0)	3 (3.9)
	Type IV	0 (0)	0 (0)	0 (0)	0 (0)	0 (0)
	Type V	0 (0)	0 (0)	0 (0)	0 (0)	0 (0)

Regarding the influence of ethnicity on the anatomical configuration of the root canals [1, 25], the results of this study can be extrapolated to the Southwestern Asian communities with caution and more extensive studies with larger sample sizes are recommended.

**Figure 1:**
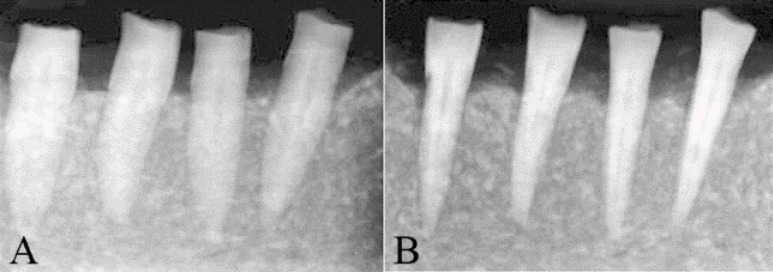
*A)* Angled and *B)* straight radiographies

Various techniques have been recommended for evaluation of the internal anatomy of the teeth *in vitro*, as stated by some authors [11, 12, 26]. According to the literature, evaluation of root cross-sections at different distances from the apex is considered as one of the assessment methods and is considered as the gold standard [27, 28].

In this investigation, root canal configuration was evaluated according to Vertucci’s classification [15, 24]. The most frequent root canal configuration for human mandibular incisors in this study was found to be Vertucci’s *type I* indicating a single canal with a single orifice ending in a single apical foramen. This configuration has been frequently observed in various investigations worldwide [16, 17, 29, 30]. 

The canal counts reported by CBCT observers was higher than those of digital radiography evaluators. This was also found in the study by Matherne *et al.* [31] who compared use of CBCT, charged coupled device (CCD) and photostimulable phosphor plate (PSP) digital radiography in identification of the root canal system. Such findings can be attributed to the fact that while observing a CBCT image, numerous and minor anatomical intricacies such as dentinal septa or calcified bridges can be indistinguishable with a separating root canal wall. This is also noteworthy that the high agreement between CBCT observers can indicate that even minor anatomical complexities can uniformly be diagnosed by different observers using this three-dimensional imaging technique. 

On the other hand, as stated previously the agreement between digital radiography observers was low which can be explained by the fact that detection of minor anatomical complexities through the two-dimensional radiographic image even with angular modifications is difficult and can be misleading even for experienced practitioners. In addition, digital radiography observers had more agreement with the results obtained by the gold standard. This can be explained by the point that endodontists observing the radiographs in two different horizontal angulations, could incorporate their clinical experience with their observations to rule out minor calcifications within the root canal system. Therefore, in determining the exact anatomical configuration of the root canal system, highly experienced observers and precise two- and, if required, three-dimensional imaging techniques are required.

## Conclusion

Although the most frequently found anatomical configuration in mandibular incisors was Vertucci’s *Type I*, the methods of evaluation did not have adequate agreement especially in more complicated canal configurations. Finally to conclude, none of the used imaging techniques per se could adequately show the exact internal anatomical configuration in mandibular incisors.
